# A Joint Energy Replenishment and Data Collection Strategy in Heterogeneous Wireless Rechargeable Sensor Networks

**DOI:** 10.3390/s21092930

**Published:** 2021-04-22

**Authors:** Mengqiu Tian, Wanguo Jiao, Yaqian Chen

**Affiliations:** The College of Information Science and Technology, Nanjing Forestry University, Nanjing 210037, China; mqtian96@njfu.edu.cn (M.T.); chencyq@njfu.edu.cn (Y.C.)

**Keywords:** wireless rechargeable sensor network, mobile vehicle, energy replenishment, data collection, UAV, limited buffer, data overflow, network lifetime

## Abstract

In wireless rechargeable sensor networks, mobile vehicles (MVs) combining energy replenishment and data collection are studied extensively. To reduce data overflow, most recent work has utilized more vehicles to assist the MV to collect buffered data. However, the practical network environment and the limitations of the vehicle in the data collection are not considered. UAV-enabled data collection is immune to complex road environments in remote areas and has higher speed and less traveling cost, which can overcome the lack of the vehicle in data collection. In this paper, a novel framework joining the MV and UAV is proposed to prolong the network lifetime and reduce data overflow. The network lifetime is correlated with the charging order; therefore, we first propose a charging algorithm to find the optimal charging order. During the charging period of the MV, the charging time may be longer than the collecting time. An optimal selection strategy of neighboring clusters, which could send data to the MV, was found to reduce data overflow. Then, to further reduce data overflow, an algorithm is also proposed to schedule the UAV to assist the MV to collect buffered data. Finally, simulation results verified that the proposed algorithms can maximize network lifetime and minimize the data loss simultaneously.

## 1. Introduction

Wireless sensor networks (WSN) have applications in many situations, for example, in smart homes, military reconnaissance, etc. [1,2,3]. However, the batteries in sensor nodes in WSNs are too small to maintain continuous operation, which poses a great challenge to the WSN [4]. To sustain the lifetime of a sensor node, it should be replaced with a new one manually. The networks are usually deployed in uninhabited areas; therefore, the implementation of such a solution is very challenging [5]. With the development of technology, wireless rechargeable sensor networks (WRSNs) which contain rechargeable sensor nodes provide a promising solution to the energy shortage problems of the WSNs [6].

There are two ways to provide energy to rechargeable sensor nodes in a WRSN [7,8]. The first is energy harvesting, which utilizes natural resources such as wind energy, solar energy, heat energy, etc. [9,10]. These kinds of natural energy are infinite and green, and have been studied by many researchers. However, such energy supplies depending on the natural environment are variable and unpredictable, which may result in the instability of the whole network [11]. Different from the energy harvesting, the second solution based on wireless energy transfer technology (WET) is stable and efficient. Industrial experiments have shown that a power of 60 W can wirelessly light a lamp with 75% energy transfer efficiency within a limited distance [12]. The efficiency and stability of this charging solution have been proven in [13]. With periodic energy replenishment of the MV, the network lifetime can be improved. To achieve the continuous operation of WRSNs, researchers have proposed many efficient charging strategies for the MV [14,15,16,17,18], which can prolong the lifetime of the network. However, the energy efficiency of data transmission has not been taken into consideration.

Most energy of a certain sensor node is consumed by transmitting sensed data to the base station [19]. If the energy of data transmission can be reduced, the urgency of charging requirements from a sensor node is relaxed, and more sensor nodes can wait until the MV charges them. In [20,21], the MV is also used to collect sensed data from network to reduce energy consumed on the data transmission. These studies showed that the network lifetime can be prolonged efficiently.

Recently, authors in [22] utilized the MV as the energy provider and the data collector simultaneously. Energy replenishment and data collection work at different frequencies; therefore, no interference exists. Many studies have developed mobile vehicle scheduling strategies to achieve efficient combinations of energy replenishment and data collection [22,23,24,25,26]. Corresponding results have indicated that the network performance could be improved significantly. However, the MV needs a certain amount of time to charge and collect data values one by one. The buffer of each sensor node is different and limited. Hence, at some time point, there may be a certain sensor node whose buffer is full, although no MV can come and collect its buffer data due to energy replenishment. This case will result in data overflow and the quality of service cannot be satisfied.

To avoid data overflow, utilizing another vehicle (only a “collect data vehicle”, CDV) which is responsible for assisting the MV in collected buffered data, is a promising solution. There is some work on collection policies of CDVs. The buffering of sensor nodes is limited, e.g., to 1024 KB [27], and sense rate of each sensor node is assumed as 10 kbps [28]; therefore, the maximum buffer time of a sensor node is about 12 min. At the same time, the weight of a CDV is relatively great, which means that traveling itself will use much energy, up to 0.6 KJ/m [25]. When the battery capacity of an MV is 2000 KJ [14], and the speed of the MV is 5 m/s [25], the travel time can only last $\frac{2000\;\mathrm{KJ}}{0.6\;\mathrm{KJ}/m\;*\;5\;m/s}=680\;s$ per tour. Hence, the total service time (charging time and collecting time) of the MV during one period is relatively shorter than the time that sensor node can wait. In other words, for a sensor node with a limited buffer, the CDV may not be able to collect data in a timely manner. Furthermore, influenced by complex road environments in remote areas, a CDV will consume more time and energy in traveling. This will further aggravate the time pressures caused by the long time which the sensor node has to wait. The longer the sensor node waits, the greater the probability of buffer data loss. Therefore, considering practical environments and the limit of CDVs, data overflow may be very serious in some scenarios if the network just uses a CDV to assist the MV in collecting buffered data.

Recently, UAV-enabled data collection, which avoids complex road environments and has higher speed and lower traveling costs, is becoming more and more popular [29,30]. Due to its inherent attributes, such as high flexibility, high mobility, and adaptive altitude, UAVs have been proven as an efficient solution to collect data from these networks [31]. Furthermore, because a UAV can hover at some altitude above the ground, it can also provide reliable downlink and uplink communications [32]. When suffering from serious natural disasters, [33] showed that, instead of communication vehicles which are heavy and greatly affected by bumpy roads, using UAVs is more likely to keep local networks connected in a cost-effective way. In practical business, one company—DJI UAV—has shown that the battery capacity of a normal UAV is 5870 mAh, the speed of a UAV can reach 15 m/s, and the longest flight of 30 min can be achieved [34]. This description indicates that the performance of a UAV in data collection is better than that of terrestrial vehicles, especially when road conditions are complex. If used to assist data collection of an MV, data overflow caused by inherent limitations of CDVs, which include slow speeds, restrictions by the environment, and short effective working times, could be further reduced.

**Figure 1:**
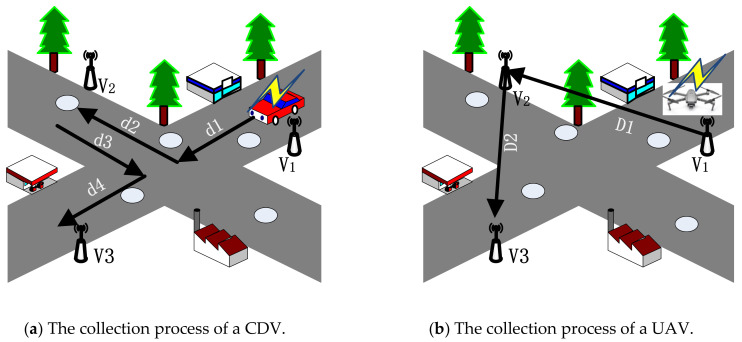
The difference in data collection performance between a CDV and the UAV.

Here, an example in Figure 1 shows the difference of data collection performance between the CDV and the UAV in practical driving environments. Assuming that three sensor nodes have buffer data which need to be collected and the collection order is $v_{1}\to v_{2}\to v_{3}$: for the CDV, because the path is paved in advance, the traveling distance of the CDV is $d_{CDV}=d_{1}+d_{2}+d_{3}+d_{4}$ and the traveling time of CDV is $t_{CDV}=\frac{d_{CDV}}{v_{CDV}}$. At the same time, in Figure 1a, the circle on the road indicates that the road is not smooth, which means that the actual traveling time will be greater than $t_{CDV}$; for the UAV (Figure 1b), because it can fly at a certain distance from the ground in the air to avoid the interference of ground obstacles, the traveling distance is $d_{UAV}=D_{1}+D_{2}$ and the travel time of the UAV is $t_{UAV}=\frac{d_{UAV}}{v_{UAV}}$. $v_{CDV}<v_{UAV}$, $d_{UAV}<d_{CDV}$; therefore, it can be easily determined that $t_{UAV}<t_{CDV}$, which means that the UAV can more quickly reach the sensor node which needs to send buffer data and the data overflow can be reduced.

To prolong the network lifetime and reduce data overflow, we propose a novel combined model of an MV and UAV. The MV is utilized to provide energy and collect buffer data of sensor nodes simultaneously. At the same time, as the auxiliary data collector of the MV, the UAV is responsible for collecting as much as data possible to further reduce the data loss. To improve the charging efficiency, sensor nodes are grouped into different clusters according to the cellular structure, and sensor nodes in the same cell are assumed as a cluster. The MV adopts a multiple-node charging method to charge all sensor nodes in the same cluster simultaneously. At the same time, to improve the collection efficiency, sensor nodes send buffer data to the cluster head node (CHN). Then, the CHN sends collected data and data buffered by itself to the MV or the UAV directly. Note that the time spent on collecting, $\frac{1024\;KB}{62.5\;kb/s}\approx 16\;s$ [18], is much less than charging time, $\frac{10.8\;KJ}{5\;Watts}=36\;\min$ [24]; we also assumed that the MV could collect buffer data from neighboring clusters to improve the total amount of collected data.

Under the novel proposed model, optimal scheduling strategies of the MV and the UAV were studied. First, the problem of maximizing the network lifetime was investigated. We use a metric named as normalized dead time to measure the network lifetime. The problem of maximizing the network lifetime was turned into minimizing the total normalized dead time of sensor nodes. Then, the problem of minimizing the amount of data loss was studied. A metric named as the amount of collected data was used to represent the amount of data which are collected by the MV or the UAV. The problem of minimizing the data loss can be transformed into maximizing the amount of collected data. The problems considered in this paper can be summarized as follows: (i) How can the MV be scheduled to charge clusters and collect buffer data so that the normalized dead time can be minimized and the amount of collected data can be maximized during the charging tour?; (ii) How can the UAV be scheduled to assist the MV in collecting buffer data from the network to maximize the amount of collected data? To deal with the above challenges, efficient algorithms are proposed.

In this paper, our main contributions can be concluded as follows:

- Considering the practical network environment (bad road condition and heterogeneous sensor nodes) and limitations of the CDV, to prolong the network lifetime while reducing the data overflow, we propose a novel model. The MV powers clusters and collects buffer data, while the UAV is the assistant data collector of the MV and responsible for collecting data from the whole network as much as possible;
- Under the new model, to maximize the network lifetime and improve the amount of collected data, an efficient joint charging and collecting algorithm (EJCCA) is proposed to schedule the MV to charge clusters and collect data from clusters needing charging and neighboring clusters;
- After determining the trajectory of the MV, considering the packet arrival rate, the amount of buffered data and the limited buffer comprehensively, an efficient data collection algorithm (EDCA) for the UAV is also proposed to further improve the network performance.

The remaining parts are organized as follows. Section 2 introduces some related work on mobile energy replenishment, mobile data collection, and a joint energy replenishment and data collection. The system model is given in Section 3, which contains the network model, the novel charging and collecting mode, the data flow routing and the energy consumption, and the problem formulation. To minimize the normalized dead time and maximize the amount of collected data from neighboring clusters, Section 4 presents an efficient joint charging and collecting algorithm. Then, a scheduling strategy of the UAV is proposed in Section 5 to further improve the amount of collected data and reduce the data loss. Section 6 analyzes the performance of proposed algorithms. Finally, this paper is concluded in Section 7. The main notations used in this paper are listed in Table 1, and a list of abbreviations is shown in Table 2.

## 2. Related Works

In this section, some current work on mobile energy replenishment, mobile data collection, and joint energy replenishment and data collection is presented.

### 2.1. Mobile Energy Replenishment

In [14], through charging sensor nodes partially, more sensor nodes needing charging can be charged in each charging period. To maximize the lifetime of the network, the optimal charging order was determined. Then, through re-scheduling the charging sequence of sensor nodes, the total traveling distance was minimized while the network lifetime could be ensured. To improve the whole charging efficiency, considering the residual energy and energy consumption rate, Ref. [15] proposed an uneven cluster structure. Then, the corresponding charging path of the MV was determined through the shortest Hamilton Cycle. To improve the charging performance of the MV, Ref. [16] proposed an efficient scheduling scheme for on-demand mobile charging. Then, the sensor nodes needing charging were reached through comprehensively considering the residual energy, the distance to the base station, and critical node density. When the size of the network increases, more sensor nodes will send charging requests to the MV, while charging only one sensor node may not satisfy so many charging demands, and the performance of the network will be influenced. To deal with this problem, Ref. [17] studied multi-node wireless energy transfer technology. Through charging all sensor nodes in a cluster at one time, the whole charging efficiency of the MV was improved significantly. Based on the charging range of the MV, a cellular structure was applied in clustering. Through jointly optimizing the traveling distance of the MV, flow routing, and charging time, an optimal charging strategy was found. In our previous work [18], through analyzing the different characteristics of different sensor nodes which were deployed in different areas, two different charging methods, single-node-charging and multiple-node-charging, were utilized comprehensively. At the same time, the optimal charging strategy was attained through a minimum matching algorithm, such that the normalized dead time of a sensor node could be minimized. However, these previous studies did not take the energy efficiency of data transmission into consideration, which is unreasonable.

### 2.2. Mobile Data Collection

Through collecting sensed data of sensor nodes by mobile vehicles, the lifetime of the network can be prolonged substantially. The reason behind this is that the energy consumed in data transmission can account for more than half of the total consumed energy, and the consumed energy will increase with the increase in the distance between the data transmitter and data receiver, while the utilization of the mobile data collector can turn the long-range communication into short-range communication. Through considering the practical situations where the network may be deployed in isolated urban areas, Ref. [20] proposed an efficient data collection algorithm to maximize the network lifetime. Firstly, the cluster head node (CHN) responsible for the data collection of all nodes in the same cluster was defined. Then, the size of each cluster was determined according to the distance to the traveling trajectory of the MV and the CHN of each cluster was dynamically elected according to the corresponding residual lifetime. Finally, the MV will collect sensed data from CHNs one by one. To achieve an optimal tradeoff between the travel path and the data transmission, the authors in [21] demonstrated a joint design of mobility control and space-decision multiple access technology. According to simulations in the above studies, it was found that the network lifetime could be improved significantly.

### 2.3. A Joint Energy Replenishment and Data Collection

To achieve continuous operation of the WRSN and the higher energy efficiency of data transmission of sensor nodes, more and more researchers have proposed the idea of using the MV as an energy provider and data collector simultaneously. Authors in [22] utilized two different types of mobile vehicles as an energy provider and data collector. Through optimizing the data rate of each sensor node, a distributed algorithm was designed and then the total network utility was maximized. Conversely, Refs. [22,23] used several mobile vehicles to charge sensor nodes which sent charging requests, and the vehicles collected sensed data simultaneously. The anchor point, which is also seen as the cluster head node, was dynamically selected according to the semi-Markov energy prediction model. Then, to minimize the travel cost, the mobile vehicles traveled along the shortest Hamiltonian path to realize the energy replenishment and the data collection from the anchor point in each cluster. Utilizing the cellular structure to divide the network into the same cells which were defined as clusters of the network, [24] determined the cluster head node through considering the energy consumption rate. Then, the mobile vehicles would travel along the optimal path, which was achieved using a discrete firework algorithm periodically, so that the amount of data collected by the unit energy of the vehicle was maximized. To maximize the network lifetime and the amount of collected data, the authors in [25] developed a mathematical model to address the problem. Then, through turning the problem into a special sub-problem which only involved space-dependent variables, the authors proposed a provably near-optimal solution. In [26], the authors used a new combined recharging and collecting data model to set up a WRSN. A k-means algorithm was used to group sensor nodes into clusters. Then, by reducing the number of dead nodes according to the residual lifetime and the location, the charging order of clusters needing charging could be determined. Mobile vehicles would travel along the predefined way to charge nodes and collect data. Previous work on joint energy replenishment and data collection has improved the performance of the network significantly.

## 3. System Model

In this section, we first introduce the system model, which contains the network model, the novel charging and collecting model and data flow routing, and the energy consumption. Then, the normalized dead time minimization problem and the data loss minimization problem are formulated, respectively.

### 3.1. Network Model

**Figure 2:**
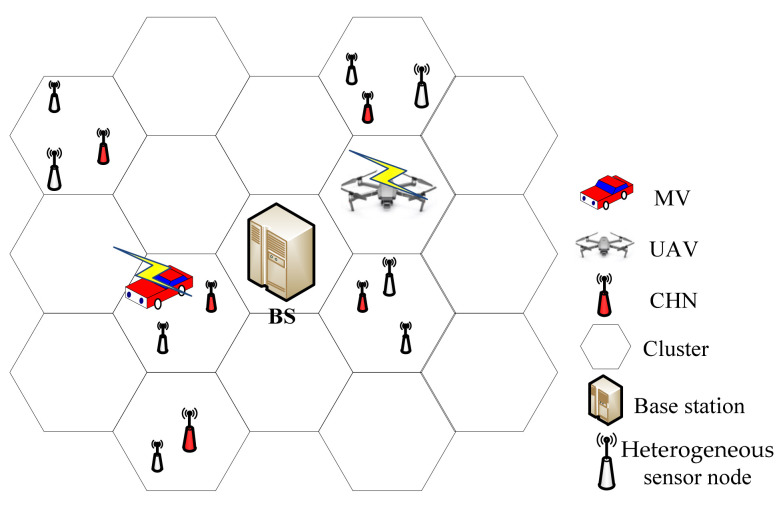
Diagram of our network.

The set V (|V|=n) is defined to represent the set of heterogeneous sensor nodes which are deployed in the network area L×L randomly. The battery capacity of each sensor node $v_{i}\in V$ is defined as $\alpha B_{\max}$, where $B_{\max}$ is the maximum battery capacity. For different sensor nodes, α is a random value selected from the interval [0.5,1], which means that sensor nodes in the network have different battery capacities. $B_{i,t}$ is used to denote the residual energy of sensor node $v_{i}$ at time t. It can be observed that $B_{i,0}=\alpha B_{\max}$. $\rho_{i}$ is the energy consumption of sensor node $v_{i}$. The residual lifetime of $v_{i}$ can be defined as $\frac{B_{i,t}}{\rho_{i}}$. The buffer of sensor node $v_{i}$ is also different and is defined as ϕ∗BU (0.5≤ϕ≤1), where BU is the maximum buffer. The coordinate of each sensor node can be written as $\left(x_{i},y_{i}\right)$. The base station S is located in the center of the network and is responsible for receiving and processing the data from the MV or the UAV. Assuming that the battery capacity of the base station S is infinite, $\varphi B_{\min}(0.5\leq \varphi \leq 1)$ can be used to represent the least energy level of each sensor node, where $B_{\min}$ is the minimum energy level. To make charging and collection more efficient, the network is divided into clusters based on the cellular structure in [18], and the CHN is dynamically selected according to the residual lifetime and the location during each period (which will be discussed later). Other sensor nodes will send their buffer data to the CHN in the same cluster, and then the CHN transmits the whole dataset to the MV or the UAV or the base station. The set of all clusters is defined as C. An example is shown in Figure 2.

### 3.2. The Novel Charging Model

We used $C_{1}=\{c_{1},c_{2},\ldots c_{num}\}$ to represent the set of clusters needing charging, where $|C_{1}|=num$ and num is the number of clusters sending charging requests. When there is a sensor node whose residual lifetime is less than the charging threshold, it will send a charging request (including information such as the residual energy, energy consumption, buffer rate, etc.) to the base station through multiple hops. Once receiving the charging request, the base station will dispatch the MV and the UAV to travel along the predefined route (the details will be discussed in Section 4.1.) to provide power to these clusters needing charging [15]. Furthermore, this information request is very small; therefore, the transmission energy can be ignored. The travel path of the MV forms a closed cycle: $S\to c_{1}\to c_{2}\to \cdots \to c_{num}\to S$. Note that sensor nodes are charged in clusters with multi-node charging technology [18]. The charging time $\tau_{c,j}$ of the MV at the $j^{th}$ cluster $c_{j}\in C_{1}$ can be denoted as:

(1) $$\tau_{c,j}=\sum_{i=1}^{cn_{j}}\frac{B_{i,0}^{j}-B_{i,t}^{j}}{U_{i,j}}$$

where $cn_{j}$ represents the number of sensor nodes in the $j^{th}$ cluster. $B_{i,0}^{j}$ is the initial energy of the $i^{th}$ sensor node in the $j^{th}$ cluster, and $B_{i,t}^{j}$ is the residual energy of the $i^{th}$ sensor node in the $j^{th}$ cluster at time point t. $B_{i,0}^{j}-B_{i,t}^{j}$ is the energy needed by the $i^{th}$ sensor node, and $U_{i,j}$ is the charging rate, which is related to the distance between the MV and the corresponding $i^{th}$ sensor node. Then, the total charging time is $\tau_{ch\arg e}=\sum_{j=1}^{num}\tau_{c,j}$.

In this paper, the CHN is responsible for transferring buffer data of all sensor nodes in the same cluster and consumes much more energy compared with other sensor nodes. The charging efficiency of the MV is related to the distance; therefore, to balance the energy load, the MV is assumed to stop at the location of the CHN. If the practical road conditions are considered, the travel distance $D_{j,j+1}$ of the MV between the $j^{th}$ cluster and $j+1^{th}$ cluster can be expressed as:

(2) $$D_{j,j+1}=l||(x_{j+1}-x_{j}),(y_{j+1}-y_{j})|{|}_{2}.$$

where l is the coefficient representing the path condition; for example, when the road is very smooth, l=1. Otherwise, 1<l<5. $\left(x_{j},y_{j}\right)$ are the coordinates of the CHN in the $j^{th}$ cluster.

$\tau_{path,j}$ is denoted as the corresponding travel time of the MV between the $j^{th}$ cluster and $j+1^{th}$ cluster, which can be expressed as:

(3) $$\tau_{path,j}=\frac{D_{j,j+1}}{v_{c}}$$

where $v_{c}$ is the speed of the MV. Then, the total travel distance of the MV is $\tau_{path}=\sum_{j=1}^{num}\tau_{path,j}$.

Therefore, the total time τ of the MV spent on a charging period can be written as:

(4) $$\tau =\tau_{ch\arg e}+\tau_{path}$$

The travel time is much shorter than the charging time; therefore, a constant Δtravel=60 s is used to represent the average travel time between two adjacent clusters needing charging [14]. Through adjusting the charging power of the MV in each cluster, the charging time of the MV spent on each cluster can be seen as the same. Then, the total time can be divided into many time slots ξ with the same length, which can be denoted as $\xi =\xi_{2}=\xi_{3}=\cdots =\xi_{num}$.

The normalized dead time $\eta_{i,j}$ of sensor node $v_{i}$ in the $j^{th}$ cluster can be defined as follows:

(5) $$\eta_{i,j}=\left\{\begin{array}{l} 0\;\;\;\;\;\;\;\;\;\;\;\;\;\;\;\;\;\;\;\;\;\;\;\;,\;\frac{l_{i,t}}{\xi }-T_{k,i}+1\geq 0\;\\ |\frac{l_{i,t}}{\xi }-T_{k,i}+1|\;,\;\frac{l_{i,t}}{\xi }-T_{k,i}+1<0\; \end{array}\right.$$

where $l_{i,t}$ is the residual lifetime of sensor node $v_{i}$ at time point t. $\frac{l_{i,t}}{\xi }$ is the number of timeslots that sensor node $v_{i}$ can survive, T is the set of charging timeslots, and $T_{k,i}$ represents sensor node $v_{i}$ being charged by the MV in the $k^{th}$ timeslot. When $\frac{l_{i,t}}{\xi }-T_{k,i}+1$ is not less than 0, it means that sensor node $v_{i}$ can operate normally before the MV powers it. Otherwise, sensor node $v_{i}$ will be out of energy before the MV powers it, and the number of dead timeslots will be $\left|\frac{l_{i,t}}{\xi }-T_{k,i}+1\right|$. The sum of normalized dead time in the $j^{th}$ cluster is $\eta_{j}=\sum_{i=1}^{nc_{j}}\eta_{i,j}$. Thus, the sum of normalized dead time $\eta_{sum}$ of the whole network during the period can be defined as:

(6) $$\eta_{sum}=\sum_{j=1}^{num}\eta_{j}$$

where num represents the number of clusters needing charging during the charging period.

According to the above discussion, the lifetime maximization problem can model the normalized dead time minimization problem. The solution of ascertaining the optimal charging order of clusters can be turned into the determination of the optimal charging timeslot selection.

### 3.3. The Novel Collecting Model

When the MV charges clusters which have sent charging requests, it can also collect buffer data from clusters to reduce the data loss and energy consumed in long-distance communications. The time $\sigma_{c,i}$ spent on data collection of the MV at sensor node $v_{i}$ can be represented as:

(7) $$\sigma_{c,i}=\frac{BD_{i,t}}{G_{MV}},$$

where $BD_{i,t}$ is the total amount of buffered data of sensor node $v_{i}$ at time t, while $G_{MV}$ is the data collection rate of the MV, e.g., 500 kb/s [24]. As described above, because the collecting time is much less than the charging time, during the charging process for the current charging cluster, the MV can also collect buffer data from neighboring clusters. Therefore, the amount of collected data can be improved, while the lifetime of the WRSN is ensured.

**Figure 3:**
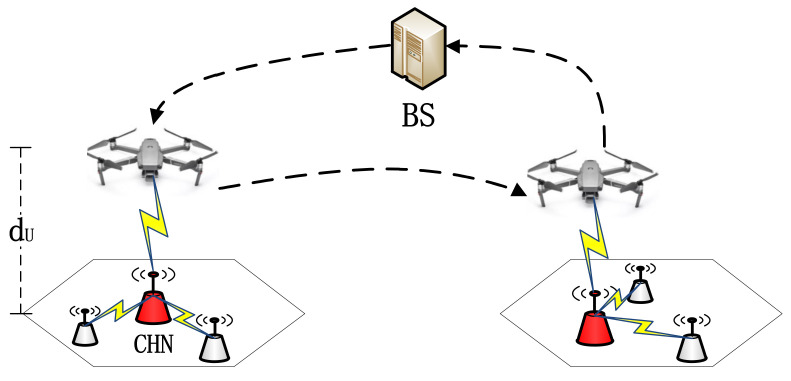
The detailed data collection process of UAV.

To assist the MV in collecting as much buffer data as possible from the network and reducing the data overflow, the UAV with light and flexible features can further be utilized. When the MV leaves the base station to power sensor nodes, the UAV also flies away from the base station to collect buffer data along the predefined route as an assistant data collector to the MV. To avoid interference from ground objects, we assumed that the UAV hovers $d_{U}$ meters above the ground, where $d_{U}$ is a relatively large number (e.g., 50 m [30]). A directional antenna is deployed on each CHN; the UAV and each CHN can communicate through an LET wireless link at certain time points [35]. To simplify the model, we assume that the data collection rate $G_{UAV}$ of the UAV is the same as that of the MV, which can be expressed as $G_{MV}=G_{UAV}=G=500\;kb/s$. The time spent on collecting data from clusters can refer to (7). Note that because there are no obstacles in the UAV’s trajectory, the travel distance $D_{j,j+1}^{UAV}$ of the UAV between the $j^{th}$ cluster and $j+1^{th}$ cluster can be expressed as $D_{j,j+1}^{UAV}=||(x_{j+1}-x_{j}),(y_{j+1}-y_{j})|{|}_{2}$. The speed of the UAV $v_{u}$ is larger than that of the MV $v_{c}$, and the corresponding travel time can refer to (3). The data collection process of the UAV is shown in Figure 3.

It is worthwhile pointing out that the buffering of sensor node is different and limited. Defining $loss_{i}$ as the data loss of the $i^{th}$ sensor node in the $j^{th}$ cluster, the data loss of the $j^{th}$ cluster can be written as:

(8) $$loss_{j^{th}cluster}=\sum_{v_{i}\in j^{th}cluster}loss_{i},$$

The total data loss of all sensor nodes can be written as:

(9) $$loss_{sum}=\sum_{v_{i}\in V}loss_{i}.$$

### 3.4. The Data Flow Routing and Energy Consumption

$f_{j,M}$ is used as the flow rate from $j^{th}$ CHN to the MV or to the UAV. For simplicity, in this sub-section, we use the MV as example, respectively. The flow balance can be expressed as:

(10) $$\sum_{k\in j^{th}cluster}^{k\neq CH_{i}}f_{k,CH_{j}}+R_{j}=f_{j,M}$$

where $R_{i}(bit/s)$ is the average sensing data rate of the $j^{th}$ cluster, and $CH_{j}$ represents the CHN of the $j^{th}$ cluster.

In this paper, energy consumed on sensing, receiving, and transmitting are considered. The practical consumption model in [19] is adopted. The energy consumption rate $\rho_{i}$ of each sensor node can be written as follows:

(11) $$\rho_{i}=\left\{\begin{array}{l} \gamma R_{i}+C_{i,M}\sum_{v_{i}\in V}^{j\neq m}f_{i,M}+\iota \sum_{v_{k}\in m^{th}cluster}^{k\neq i}f_{k,i}v_{i}\;\;is\;\;CHN\\ \gamma R_{i}+C_{i,}{}_{CH_{m}}f_{i,}{}_{CH_{m}}\;\;\;\;\;\;\;\;\;\;\;\;\;\;\;\;\;\;\;\;\;\;\;\;\;\;\;\;\;\;\;\;\;\;\;\;\;\;\;\;\;\;\;\;\;\;\;\;\;\;\;\;else, \end{array}\right.$$

where sensor nodes are divided into two types, CHN and normal sensor node. As described above, the CHN is responsible for collecting all data from other sensor nodes in same cluster and transmitting them to the MV. Therefore, the energy consumption of the CHN includes sensing, receiving, and transmitting activities. If sensor node $v_{i}$ is the CHN of the $m^{th}$ cluster, $\gamma R_{i}$ is the energy consumption rate of $v_{i}$. $C_{i,M}$ is the energy consumption rate for transmitting one unit of data from $v_{i}$ to the MV and $C_{i,M}=\beta_{1}+\beta_{2}D_{i,M}^{\alpha }$, where $\beta_{1}$ is the constant term independent of distance and $\beta_{2}$ is the constant term related to the distance. $\iota \sum_{v_{k}\in m^{th}cluster}^{k\neq i}f_{k,i}$ is the energy consumption rate of sensor node $v_{i}$ for receiving data from others in the $m^{th}$ cluster. For the normal sensor node $v_{i}$, its energy consumption only contains sensing and transmitting.

Under the novel model proposed in Section 3.2, the CHN can be divided into three kinds: (i) CHN being charged and sending data to the MV (CHN needing charging); (ii) CHN sending data to the MV (and some neighboring CHNs); and (iii) CHN being charged and sending data to the UAV. Based on such classifications, the corresponding residual energy $B_{i,t}$ of a certain CHN $v_{i}$ is shown as follows:

(12) $$B_{i,t}=\left\{\begin{array}{l} \alpha B_{\max}\;\;\;\;\;\;\;\;\;\;\;\;\;\;\;\;\;\;\;\;\;\;i\in (i)\\ B_{i,t}-\rho_{i}t\;\;\;\;\;\;\;\;\;\;\;\;\;\;\;\;i\in (\mathrm{ii}\;\mathrm{or}\;\mathrm{iii})\; \end{array}\right..$$

### 3.5. Problem Formulation

The operation of the network consumes limited energy of the sensor nodes continuously; to prolong the network lifetime and achieve high network performance, the MV is periodically needed to power sensor nodes. To improve the energy efficiency of the data transmission and reduce the data overflow, the MV should also be used to collect buffered data from the network simultaneously. The charging time is much longer than the collecting time; therefore, the MV could also be used to collect data from neighboring clusters. Then, to further reduce the data overflow, the flexible and light UAV can also be utilized as an assistant data collector of the MV to collect as much data as possible.

First, (i) the normalized dead time minimization problem is defined as follows. Given a set of clusters needing charging, the MV is scheduled to power them in clusters along the predefined route. When the time is divided into even timeslots, the problem is how to find the optimal charging timeslot sequence of each cluster to maximize the network lifetime. Then, for (ii) the data loss minimization problem, it can be turned into the amount of collected data maximization problem. According to the above description, this problem can be divided into two sub-problems: (ii–i) the amount of collected data maximization problem for neighboring clusters is defined to choose the optimal CHNs in neighboring clusters to maximize the amount of collected data $AC_{data}$ during the charging tour. (ii–ii) The amount of collected data maximization problem delivered by the UAV is defined as follows. For clusters which are not charged at some period, while considering the packet arrival rate, the amount of buffered data and the different and limited buffer in the heterogeneous WRSN, the problem is how to choose optimal CHNs to maximize the amount of collected data, so that the data loss can be minimized.

In summary, the problems considered in this paper are defined as follows:

(13a) $$\max\;\;AC_{data}$$

(13b) $$s.t.\;\;\;\;\eta_{sum}=\eta_{sum}^{*}$$

(13c) $$TN_{sum,j}+\sigma_{c,j}\leq \tau_{c,j}$$

(13d)  BU≤ψ

(13e) $$d_{U}\geq \zeta$$

The constraint (13b) ensures that the lifetime of the WRSN can be maximized, where $\eta_{sum}^{*}$ represents the minimized sum normalized dead time. (13c) ensures that the time spent on collecting the buffered data of the $j^{th}$ cluster plus that of collecting data of neighboring clusters $TN_{sum,j}$ is no more than the charging time at the $j^{th}$ cluster, so that the network lifetime can be ensured. ψ is a small constant and (13d) means that the buffering of each sensor node is limited. (13e) represents the altitude of the UAV, which should be larger than a relatively large constant ζ to avoid interference from ground objects.

## 4. An Efficient Joint Charging and Collection Algorithm

In this section, to deal with the normalized dead time minimization problem and the amount of collected data maximization problem for neighboring clusters, we propose an efficient joint charging and collection algorithm to schedule the MV to charge and collect data. Then, an optimality proof of our proposed algorithm is given.

### 4.1. Algorithm

First, the set $C_{1}$ of clusters needing charging is determined. Details of the choosing process are shown as follows. $C_{1}$ is initialized as empty: if there is a certain cluster whose residual lifetime is less than the charging threshold (a value that ensures sensor nodes of the network can be charged at the appropriate time, which is assumed as 5 h), it will be added into set $C_{1}$. Then, the time spent on clusters in $C_{1}$ in turn is subtracted from the residual lifetime of the remaining clusters; if the resultant number is no greater than the charging threshold, the corresponding cluster will also be added into the set $C_{1}$. Finally, when the set $C_{1}$ is stable, a route attending all clusters needing charging in one period can be devised.

Once clusters send charging requests, the base station will send the MV to power these clusters in an optimal charging order to maximize the lifetime of the WRSN. As mentioned above, time is divided into many identical timeslots and the problem of the optimal charging order can be turned into one of optimal charging timeslot matching. First, a bipartite graph $G=(C_{1},T,\omega ,E)$ is constructed, where the weight $\omega (c_{i},T_{k,i})$ of each edge $(c_{i},T_{k})\in E$ represents the normalized dead time when the $i^{th}$ cluster is matched with the $k^{th}$ timeslot. Based on the Kuhn–Munkres algorithm, a minimum weighted matching $M_{\min}$ of clusters needing charging and charging timeslots can be found. Then, the minimum weighted matching $M_{\min}$ can be transferred into the optimal charging order $O_{ch\arg e}$ of clusters.

After determining the travel path of the MV, the MV will charge nodes and collect data from CHNs simultaneously. In this paper, the CHN of each cluster is selected according to the location, the buffer size, and the residual lifetime comprehensively. $weight_{i,j}$ is defined as the weight value of the $i^{th}$ sensor node in the $j^{th}$ cluster, which can be defined as:

(14) $$weight_{i,j}=\varpi \frac{\sum_{m\neq i,m\in j^{th}cluster}d_{i,m}}{\sum_{i=1}^{cn_{j}}d_{i,cen}}+\theta \frac{BU_{i}}{BU_{\max}}+\vartheta \frac{B_{i,t}^{j}}{B_{\max}},$$

where $\sum_{m\neq i,m\in j^{th}cluster}d_{i,m}$ represents the total distance between the $i^{th}$ sensor node and other sensor nodes, $cn_{j}$ is the number of sensor nodes in the $j^{th}$ cluster, and $\sum_{i=1}^{cn_{j}}d_{i,cen}$ is the total distance between the center location and all sensor nodes in the $j^{th}$ cluster. $BU_{i}$ is the buffer of the sensor node, and $BU_{\max}$ is the maximum buffer among sensor nodes. $B_{i,t}^{j}$ is the residual energy of the $i^{th}$ sensor node in the $j^{th}$ cluster, and $B_{\max}$ is the maximum residual energy among sensor nodes. ϖ,θ,ϑ are the weight coefficients, where ϖ+θ+ϑ=1. The CHN is responsible for collecting buffer data from other sensor nodes and transmitting data to the base station or the MV. Hence, the energy consumption of the CHN is more than the others. To improve the probability of successful data transmission and prolonging the network lifetime, the CHN should: (i) be closer to the center of the cluster, so that energy consumed on data transmission in the cluster can be minimized; (ii) have a larger buffer, so that more data can be stored without overflowing; and (iii) have more residual lifetime, so that the work time can be longer. We consider the above three factors comprehensively in (14) and select the sensor node with maximum $weight_{i,j}$ as the CHN. The dynamic selection of the CHN in each charging period can also balance the distribution of energy load.

From (14), it can be found that different CHNs of each cluster can be achieved through adjusting three factors ϖ,θ,ϑ. When ϖ is larger, the sensor node which is closer to the center of the cluster will have more opportunity to be selected as the CHN, while the buffer and the residual energy are secondary consideration factors. If θ is larger than the other two factors, the buffer of the sensor node will play the most important role in CHN selection. When ϑ is larger, the sensor node with the most residual energy will be more competitive. In the simulation, impacts of different values of the above factors on network performance were studied.

When the MV collects buffer data from the CHN, there is one point which should be noted. The buffering of sensor nodes is limited; therefore, at some time point t, the sensor node may already have overflowed many times. Then, the amount of buffer data that the MV can collect is shown as follows:

(15) $$BD_{i,t}=\mathrm{mod}(\frac{(k-1)*\xi }{BU_{\max}/\gamma R_{i}})*\gamma R_{i}$$

where k−1 is the charging order of sensor node $v_{i}$, and (k−1)*ξ is the time when the MV arrives at sensor node $v_{i}$. mod(•) represents the remainder of •. $BU_{\max}/\gamma R_{i}$ is the maximum buffer time, while $\mathrm{mod}(\frac{(k-1)*\xi }{BU_{\max}/\gamma R_{i}})$ represents the time from the last data overflow.

The time spent on charging the current cluster may be longer than that spent collecting data; therefore, the buffered data from corresponding neighboring clusters can also be collected by the MV within the charging time. To accomplish such a procedure, the set $NE_{j}$ of neighboring clusters to the current $j^{th}$ charging cluster should be found first. The CHN $CH_{j}$ of a neighboring cluster set $NE_{j}$ should satisfy the following constraints:

(16) $$D_{CH_{j},CH_{c}}\leq RC_{CH_{j}},$$

where $CH_{c}$ is the CHN of the current charging cluster, and $RC_{CH_{j}}$ is the communication range of $CH_{j}$; it is worth pointing out that communication range of each sensor node in our network was different. $D_{CH_{j},CH_{c}}\leq RC_{CH_{j}}$ ensures that the CHN $CH_{j}$ can send buffered data to the CHN $CH_{c}$ successfully.

Then, the MV will choose the optimal neighboring clusters from which to collect data to maximize the normalized saved energy during the charging period. $\tau_{c,j}-\sigma_{c,j}$ is defined as the available time $AT_{j}$ of the $j^{th}$ cluster. According to (13c), when the MV is charging the $j^{th}$ cluster, the total time used to collect data from neighboring clusters, $TN_{sum,j}$, should be no greater than $AT_{j}$, which can be expressed as:

(17) $$TN_{sum,j}\leq AT_{j}.$$

To minimize the data overflow, the order of collection in set *NE_j_* should be optimized according to the descending volumes of buffered data, such that the amount of collected data of neighboring clusters during the limited charging period can be maximized.

More details can be found in Algorithm 1.
**Algorithm 1.** An efficient joint charging and collection algorithm.**Input**: The set V, the coordinates of the sensor node $\left(x_{i},y_{i}\right)$, the initial energy $B_{i,0}$, the residual energy $B_{i,t}$, the energy consumption $\rho_{i}$, and the buffered data $BD_{i,t}$. **Output**: The normalized sum of dead time of the network $\eta_{sum}$, the sum amount of collected data of clusters during the charging period. 1: Obtain the set $C_{1}$ of clusters needing charging and determine the CHN of each cluster according to (14). 2: According to the needed energy of each cluster, timeslots with the same length can be achieved through adjusting the corresponding charging power of the MV. 3: **for**
i←1 to $\left|C_{1}\right|$
**do** 4: Find the minimum weighted matching $M_{\min}$. 5: **end for** 6: Transfer the set $M_{\min}$ into the charging sequence of clusters $O_{ch\arg e}$. 7: **for**
i←1 to $\left|O_{ch\arg e}\right|$
**do** 8: Find the set $NE_{i}$ of neighboring clusters according to (16). 9: Re-order the sequence of neighboring clusters according to the amount of buffered data. 10:$TN_{sum,i}=0$. 11: **for**
j←1 to $\left|NE_{i}\right|$
**do** 12: **if**
$AT_{i}\leq TN_{sum,i}$
**then** 13: Break. 14: **end if** 15: $TN_{sum,i}=TN_{sum,i}+\sigma_{c,j}$.%$\sigma_{c,j}$ is the collection time of the MV at the $j^{th}$ neighboring cluster. 16: **end for** 17: **end for**

### 4.2. Algorithm Analysis

**Theorem** **1.** *Algorithm 1 can achieve an optimal solution to the normalized dead time minimization problem and the amount of collected data maximization problem for neighboring clusters.*

**Proof** **of** **Theorem** **1.** The optimal order of clusters needing charging is the solution to the normalized dead time minimization problem. In this paper, we divide the time into even timeslots, and then the optimal charging order problem can be transferred into the optimal charging timeslot matching problem. Through the Kuhn–Munkres algorithm, a minimum weighted matching $M_{\min}$ of the cluster needing charging can be found, where the sum weight $\sum_{c_{i}\in C_{1}}\omega (c_{i},T_{k,i})$ is minimized. As described above, the weight $\omega (c_{i},T_{k,i})$ represents the normalized dead time when the $i^{th}$ cluster matches the $k^{th}$ timeslot. Therefore, the sum of the normalized dead time of clusters can be minimized.

Neighboring clusters to the currently charging cluster compete to send buffered data to the MV to reduce the corresponding data loss. Given the set of neighboring clusters, the more buffered data they have, the greater amount of data can be collected. In Algorithm 1, full use of the available time *AT_i_* of the $i^{th}$ cluster is made to collect buffered data from the closer neighboring clusters in a “greedy” way. Therefore, the amount of collected data from neighboring clusters during the charging period can be maximized. At the same time, the total time spent on collecting data of neighboring clusters will not be more than the available time $AT_{i}$; thus, minimal normalized dead time can also be ensured. □

## 5. An Efficient Data Collection Algorithm

In this section, considering the practical network environment (the complex road conditions and heterogeneous sensor nodes) and the limitation of the vehicle in the data collection, as an assistant data collector, the UAV is applied to further reduce the data overflow. Then, an efficient data collection algorithm is proposed to select optimal CHNs from which to collect data to maximize the amount of collected data during the collection process by the UAV. After that, a vivid illustration of the proposed algorithm is given.

### 5.1. Algorithm

To further reduce the data overflow and improve the amount of collected data, a light and flexible UAV is to be utilized as an assist data collector. The number of UAVs is limited; therefore, to maximize the sum amount of collected data, the collection path of the UAV should be scheduled reasonably.

In this paper, to simplify the model, we assumed that when the MV travels the defined path P to charge sensor nodes and collect buffered data from the current charging cluster and neighboring clusters, the UAV assists the MV in collecting data from other clusters (the set of other clusters is defined as $C_{3}$) which are away from the path P. At the same time, energy consumed by the UAV when collecting data is ignored.

Due to the selfishness of each cluster, all will send a collecting request and want to be served by the UAV to avoid data overflow. To maximize the sum amount of collected data and the network utility, the UAV should select optimal clusters to serve and determine the collecting sequence of clusters. In this paper, to measure the collecting priority of each cluster, considering the packet arrival rate (defined as the $\gamma R_{i}$) and the amount of buffer data comprehensively, the weight value $weightU_{c,j}$ of the $j^{th}$ cluster is defined as:

(18) $$weightU_{c,j}=\mu \frac{\sum_{i=1}^{cn_{j}}BD_{i,t}}{BD_{ava}}+\nu \frac{\gamma \sum_{i=1}^{cn_{j}}R_{i}}{R_{ava}}$$

where $BD_{ava}$ is the average amount of buffered data of the CHNs at time t. Considering the limited different buffer, $BD_{i,t}$ can be calculated by Equation (15). $R_{ava}$ is the average packet arrival rate of CHNs. μ,ν are the weight coefficient, where μ+ν=1. From Equation (18), it can be observed that different sets of optimal CHNs to be attended can be achieved through adjusting μ and ν. When μ is greater than ν, the UAV will preferentially choose the CHN which has a larger amount buffered data at time t to serve. Otherwise, the CHN whose packet arrival rate is larger will be selected to avoid data loss. In the simulation, impacts of different values of the above two factors on network performance were analyzed. Clusters in set $C_{3}$ are re-ordered through the corresponding weight value in a descending way. Then, the UAV will collect buffered data from the CHNs one by one along the predefined sequence. Note that energy consumed on data collection is really small, and such consumption is ignored in this paper.

More details can be found in Algorithm 2.
**Algorithm 2.** An efficient data collection algorithm.**Input**: The set V, the coordinate of sensor node $\left(x_{i},y_{i}\right)$, the initial energy $B_{i,0}$, the residual energy $B_{i,t}$, the energy consumption $\rho_{i}$, and the buffered data $BD_{i,t}$. **Output**: The sum amount of collected data delivered by the UAV. 1: Obtain the set $C_{3}$ of clusters to be attended. 2: **for**
i←1 to $\left|C_{3}\right|$
**do** 3: Calculate the corresponding weight value according to Equation (18). 4: **end for** 5: Re-order the collection sequence of CHNs in set $C_{3}$. 6: T=0. 7: **for**
i←1 to $\left|C_{3}\right|$
**do** 8: **if**
T≤τ
**then** 9: The UAV collects the buffered data from the $i^{th}$ CHN. 10: Update $T=T+\sigma_{c,i}$. 11: **else** 12: Break. 13: **end if** 14: **end for**

### 5.2. An Example of Algorithm 2

Here, an example is utilized to illustrate the execution of Algorithm 2. Assuming that there are five clusters $\left\{c_{1},c_{2},c_{3},c_{4},c_{5}\right\}$ deployed in the network: the set $C_{1}$ of clusters needing charging only contains one cluster, $c_{1}$; the charging time is 2 s. At the same time, the neighboring cluster of $c_{1}$ is $c_{2}$. The MV could collect buffered data from cluster $c_{2}$ during the charging process. Note that set $C_{3}$ contains $c_{3},c_{4},c_{5}$. At some time t, the assumption is that $BD_{3,t}=1.5\;kb$, $BD_{4,t}=2\;kb$, $BD_{5,t}=2.3\;kb$, $BD_{ava}=2\;kb$, $\gamma R_{3}=0.1\;kb/s$, $\gamma R_{4}=0.15\;kb/s$, $\gamma R_{4}=0.02\;kb/s$, $R_{ava}=0.1\;kb/s$, and G=2 kb/s. Letting μ=ν=0.5, the weight value of each cluster can be achieved through Equation (18), such as, $weightU_{c,3}=0.5*\frac{1.5}{2}+0.5*\frac{0.1}{0.1}=0.875$. According to the corresponding weight value, the optimal sequence of clusters to be attended is $c_{4},c_{3},c_{5}$. Then, the UAV will collect buffer data along the predefined sequence.

## 6. Performance Evaluation

In this section is a report of verification of the proposed algorithms using MATLAB. The impacts of different parameters on the performance of the algorithms were analyzed, including the size of the network, the charging rate of the MV, the data collection rate of the MV and UAV, the weighting factor of CHN selection, and the weighting factor of optimal collecting priority.

### 6.1. Parameter Setting

The network was divided into many cells; each cell was assumed as a cluster and a cluster may contain 0~3 sensor nodes. We used the number $N_{cluster}$ of clusters whose sensor nodes’ number was greater than 0 to represent the network size. For example, $N_{cluster}=200$ meant that the network contained about 400~600 sensor nodes. Sensor nodes with different configurations were deployed in a 1000 m×1000 m area. The maximum battery capacity $B_{max}=10.8\;KJ$ [36]; thus, the battery capacity of each sensor node was a value randomly selected from an interval [5.4 KJ,10.8 KJ]. The data rate of sensor node $v_{i}$ was selected from an interval $\left[b_{\min},b_{\max}\right]$, where $b_{\min}=1\;\mathrm{kbps}$ and $b_{\max}=10\;\mathrm{kbps}$ [28]. The speed of the MV and the UAV were 5 m/s and 15 m/s, respectively [24,31]. The charging rate of the MV was an integer within the range [$U_{\min},U_{\max}$], where $U_{\min}=1\;\mathrm{Watt}$ and $U_{\max}=10\;\mathrm{Watts}$ [16]. To simplify the model, the data collection rate of the MV was equal to that of the UAV, which is written as $G_{MV}=G_{UAV}=G=500\;\mathrm{kb/s}$ [24]. The buffer $BU_{i}$ of each sensor node was different and less than 1024 KB [27]. This study took practical road conditions into consideration, in order to better simulate the real road conditions; path condition coefficients of different roads were positive values selected from an interval [1,5] randomly. The charging threshold was $l_{c}=5\;h$, and the monitoring period was one year. The weighting factor values of CHN selection ϖ,θ,ϑ were set as 0.5, 0.3, and 0.2, respectively, while the weighting factor values of optimal collection priority μ,ν were set as 0.7 and 0.3 by default.

Besides the proposed algorithms the efficient joint charging and collecting algorithm (EJCCA) and the efficient data collection algorithm (EDCA), other algorithms, (FA) [16] and (JA) [23], are also displayed to compare with our proposed algorithms, respectively.

### 6.2. Results and Analysis

**Figure 4:**
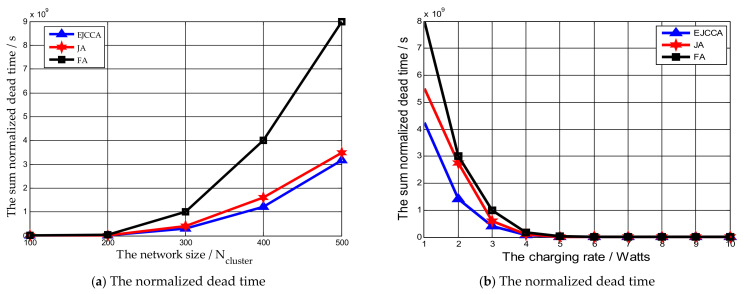
The influences of different parameters on normalized dead time.

In Figure 4a, the network size ranges from 100 $N_{cluster}$ to 500 $N_{cluster}$, while the charging rate of the MV is 5 Watts, the data collection rate is 500 kb/s, and ϖ,θ,ϑ,μ,ν are set as 0.5, 0.3, 0.2, 0.7 and 0.3, respectively. Figure 4a shows that with the increase in the network size, the sum of normalized dead time delivered by the mentioned algorithms is increased. The reason is that with the growing number of sensor nodes in the network, the number of sensor nodes needing charging is also increased. Thus, the MV may not charge them in a timely manner, which will result in the death of sensor nodes. At the same time, it should be noted that the sum of normalized dead time delivered by our proposed algorithm was less than that by other two algorithms. This is because algorithms FA and JA take the traveling distance of the MV and the residual lifetime into consideration, while our proposed algorithm just focuses on the lifetime of the network. Figure 4b indicates the impact of charging rate of the MV on the sum of normalized dead time of network. The charging rate is from 1 Watt to 10 Watts, while the network size is the $200\;N_{cluster}$ and the data collection rate is 500 kb/s. It can be found that with the increase in the charging rate, the sum of normalized dead time delivered by algorithms EJCCA, FA, and JA are reduced sharply. The reason behind this phenomenon is that more sensor nodes can be charged when the time spent on charging sensor nodes is decreased. Meanwhile, the sum of normalized dead time by our algorithm is still less than that by others.

**Figure 5:**
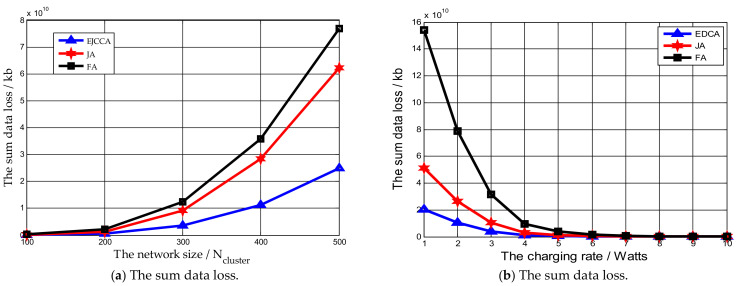
The influences of different parameters on the data loss.

The influences of different parameters on the data loss are shown in Figure 5. In Figure 5a, the impact of network size on the data loss is studied, where the network size is from 100 $N_{cluster}$ to 500 $N_{cluster}$, while the charging rate of the MV is 5 Watts, the data collection rate is 500 kb/s, and ϖ,θ,ϑ,μ,ν are set as 0.5, 0.3, 0.2, 0.7 and 0.3, respectively. Notably, the amount of data lost increased with the increase in the network size. This is easy to explain: a greater network size means that more sensor nodes will need to send buffered data, but the number of the data collectors (e.g., the MV and the UAV) is limited in practical, thus some buffer data will not be collected in time by the MV and the UAV, eventually leading to data loss. It can also be seen that the data loss by our proposed algorithm is minimal comparing with others. This is because in this study, the practical network environment is considered; the utilization of the mobile vehicle to collect buffered data in algorithm JA is not reasonable. To avoid obstacles on the traveling path, a flexible and light UAV is applied. Thus, the buffer data of sensor nodes can be collected in a faster way and the data loss of the network can be considerably reduced. Figure 5b shows the performance of algorithms by varying the charging rate from 1 Watt to 10 Watts, while the network size is $200\;N_{cluster}$ and the data collection rate is 500 kb/s. It can be observed that with the increase in the charging rate, the data loss amount is reduced. This is because with the decrease in time spent on charging each sensor node, more sensor nodes can be charged and collected during the charging period. The amount of data lost by our proposed algorithm is less than that by others. For example, when the charging rate is 3 Watts, the amount of data lost through the other simulated algorithms EJCCA, FA and JA was $4.2\times 10^{9}\;\mathrm{kb}$, $1.05\times 10^{10}\;\mathrm{kb}$ and $3.16\times 10^{10}\;\mathrm{kb}$, respectively. At the same time, it could also be found that with the increase in the charging rate, the difference between the above algorithms became smaller.

**Figure 6:**
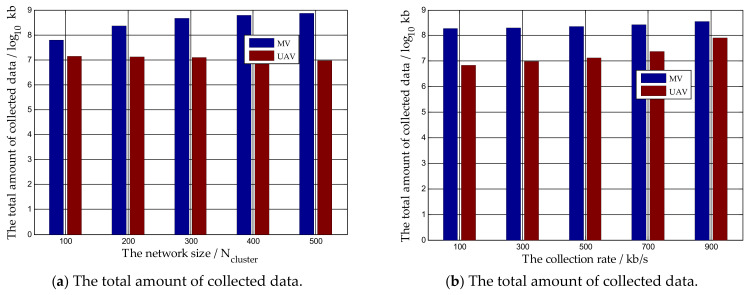
The influences of different parameters on the amount of collected data by our algorithms.

We also investigated the impacts of network size and the collection rate on the amount of collected data delivered by our proposed algorithm. In Figure 6a, the network size is from 100 $N_{cluster}$ to 500 $N_{cluster}$, while the charging rate of the MV is 5 Watts, the data collection rate is 500 kb/s, and ϖ,θ,ϑ,μ,ν are set as 0.5, 0.3, 0.2, 0.7 and 0.3, respectively. It can be found that with the increase in the network size, the amount of collected data by the MV becomes greater. Here, there is an interesting phenomenon: with the increase in the network size, the amount of data collected by the UAV decreases slightly. The reason behind this is to avoid interference between the MV and the UAV, we assume that the UAV is just responsible to collect buffered data away from the charging path, i.e., clusters which the UAV serves do not include clusters needing charging or neighboring clusters. When the network size becomes larger, more clusters will become in need of charge and more clusters will become neighboring clusters. Thus, the number of other kinds of clusters which send buffered data to the UAV will decrease. In Figure 6b, the data collection rate of the MV and the UAV range from 100 kb/s to 900 kb/s, while the network size is $200\;N_{cluster}$ and the charging rate of the MV is 5 Watts. Obviously, with the increase in the data collection rate, the amount of data collected by the MV and UAV increases.

**Figure 7:**
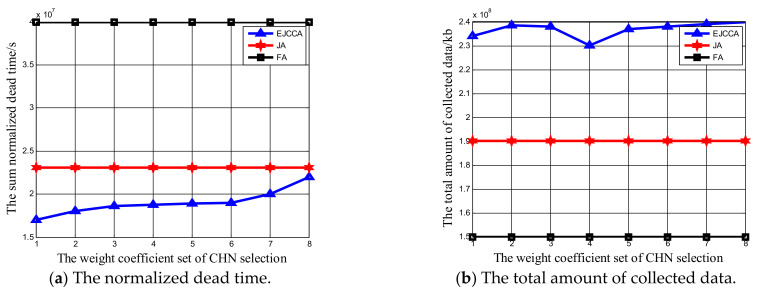
The influence of weight coefficient set of CHN selection on the network performance.

**Figure 8:**
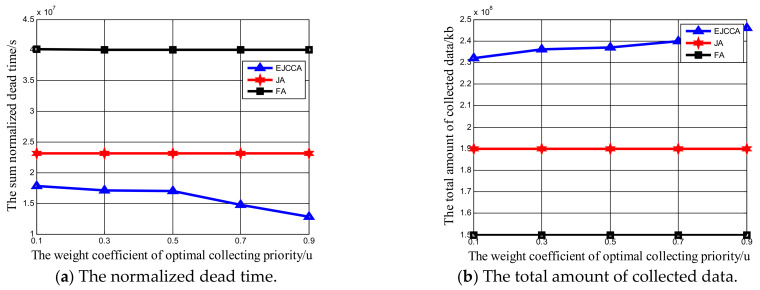
The influence of weight coefficient of optimal collecting priority on the network performance.

Impacts of five weighting factors ϖ,θ,ϑ,μ,ν on network performance were also analyzed. Notably, that there are many combinations of different sets of weighting factors. In order to simplify simulations, only some values were used for analysis. The network size was 200 $N_{cluster}$, the charging rate of the MV was 5 Watts, and the data collection rate was 500 kb/s. Furthermore, it is worthwhile pointing out that other algorithms do not use weighting factors to determine optimal CHNs or collecting priority. Hence, the normalized dead time and total amount of data collected by algorithms FA and JA are constant. First, for ϖ,θ,ϑ, different values are listed in Table 3, while the weight coefficients of optimal collecting priority, μ,ν, are 0.7 and 0.3, respectively. From Figure 7a, it can be determined that when the weight coefficient set of CHN selection is 1, i.e., ϖ,θ,ϑ are 0, 0 and 1, respectively, the sum of normalized dead time delivered by our proposed algorithm reaches the minimum value, $1.7\times 10^{7}\;s$. The reason is that the residual energy plays a more important role in CHN selection than other factors. A sensor node with more energy will be selected as the CHN. The energy consumption of the CHN is greater than other sensor nodes in same cluster; because the CHN has enough energy, the heavy data transmission work will not cause the early death of it. At the same time, when the weight coefficient set of CHN selection is 8, i.e., ϖ,θ,ϑ are 0, 1 and 0, respectively, the sum of normalized dead time reaches the maximum value, $2.2\times 10^{7}\;s$. This is because in the selection of the CHN, the buffering of the sensor node plays the most important role. Hence, the selected CHN will have a larger buffer but less residual energy. Heavy data transmission will lead to early energy depletion. At the same time, with the increase in x label, it can be found that the sum normalized dead time with our algorithm also increased. The weighting factor ϑ decreases; therefore, a certain sensor node whose residual energy is not the highest will be selected as the CHN. It is also obvious that with the increase in x label, the normalized dead time delivered by the proposed algorithm EJCCA will always be less than that by algorithms JA and FA. In Figure 7b, the total amount of collected data is maximum when the weight coefficient set of CHN selection is 8, which means that more data can be collected when ϖ,θ,ϑ are set as 0, 1 and 0, respectively. The reason is the chosen CHN has larger buffer, and more data can be cached before the data collector comes. Meanwhile, note that the total amount of collected data by set four is less than that by set one. This is because the normalized dead time by set one is less than that by set four, which means that the data collector can collect data for a longer time. When the sequence of the x label is 8, 7, 2, 6, 3, 5, 1, 4, it can be observed that the total amount of collected data decreased monotonously. The reason is that when the sequence of the x label is 8, 7, 2, 6, 3, 5, 1, 4, the weighting factor θ is also decreased, which means that less data can be buffered before the mobile data collator arrives. Meanwhile, note that the performance of our algorithm was always better than that of others when the weighting factors varied. Then, for μ,ν, μ ranges from 0.1 to 0.9 and ν ranges from 0.9 to 0.1 accordingly, while the weight coefficients of CHN selection are 0.5, 0.3 and 0.2, respectively. From Figure 8a, it can be found that with the increase in the weighting factor μ and the decrease in the weighting factor ν, the sum of normalized dead time is decreased. Moreover, the normalized dead time achieved by the proposed algorithm EJCCA was always less than that by algorithms JA and FA. At the same time, in Figure 8b, with the increase in the weighting factor μ and the decrease in the weighting factor ν, the total amount of collected data was also increased. The reason behind the above phenomenon is that the buffer is the main factor which influences optimal collecting priority. When clusters have a larger buffer, more data can be buffered and collected. Meanwhile, more data collected by the data collector means less energy consumed performing long-range data transmissions. Hence, the normalized dead time can be reduced. Meanwhile, it is worth pointing out that when the weight coefficient of optimal collecting priority μ varies, the total amount of collected data delivered by our algorithm is always better than other algorithms.

.

## 7. Conclusions

In this paper, a heterogeneous WRSN utilizing an MV and a UAV to charge nodes and collect buffered data was studied. Considering the limited buffer of each sensor node and the practical network environment, a novel data collection technique was proposed to maximize the network lifetime and minimize the data loss. The MV was responsible for powering sensor nodes and collecting data in clusters. As the assistant data collector to the MV, the UAV collected data from the network as much as possible. Under the proposed novel model, two optimal problems were formulated: minimizing the normalized dead time, and maximizing the amount of collected data. The latter problem can be divided into two sub-problems, which are maximizing the amount of collected data of neighboring clusters, and maximizing the amount of collected data delivered by the UAV. Efficient algorithms have also been proposed to solve these problems. Firstly, based on even timeslots, an efficient algorithm was proposed to maximize the network lifetime through determining the optimal charging sequence of clusters needing charging. The charging time is usually larger than the collecting time; therefore, to further improve the amount of collected data, the optimal neighboring clusters to send buffered data to the MV were also determined. Considering the packet arrival rate, the amount of buffered data, and the different and limited buffers comprehensively, the optimal set of clusters which could send buffered data to the UAV could be determined. Then, the amount of collected data delivered by the UAV was maximized. Finally, the proposed algorithms were verified by MATLAB; the results indicated that network performance could be improved by algorithms in this paper.

Furthermore, it is worthwhile pointing out that the number of MVs and UAVs are open issues. In the future, the impacts of different numbers of them in the network performance will be studied.

## Figures and Tables

**Table 1:** Symbol definitions.

Symbol	Definition	Symbol	Definition
$B_{\max}$	The maximum battery capacity.	$\eta_{sum}$	The sum of normalized dead time of the whole network
V	The set of heterogeneous sensor nodes.	$\eta_{c}$	The sum of normalized dead time of the $c^{th}$ cluster.
$\rho_{i}$	The energy consumption of sensor node $v_{i}$.	$\sigma_{c,j}$	The time spent on data collection by the MV at the $j^{th}$ cluster.
$l_{i,t}$	The residual lifetime of sensor node $v_{i}$ at time t.	$BD_{i,t}$	The total amount of buffered data of sensor node $v_{i}$ at time t.
$T_{k,i}$	The charging order of sensor node $v_{i}$	$BD_{ava}$	The average amount of buffered data of CHNs at time t.
num	The number of clusters sending charging requests.	ε	The coefficient representing different battery capacities of sensor nodes.
BU	The maximum buffer of sensor node.	$\beta_{1}$	The constant term independent of distance.
C	The set of all clusters.	$\beta_{2}$	The constant term related to the distance.
$C_{1}$	The set of clusters needing charging.	$G_{MV}$	The data collection rate of the MV.
$B_{\min}$	The minimum energy level needed to keep some sensor node working.	$loss_{i}$	The data loss of sensor node $v_{i}$.
$\tau_{c,j}$	The charging time of the MV at the $j^{th}$ cluster.	$B_{i,t}$	The residual energy of sensor node $v_{i}$.
$cn_{j}$	The number of sensor nodes in the $j^{th}$ cluster.	$AC_{data}$	The amount of collected data.
$B_{i,0}^{j}$	The initial energy of the $i^{th}$ sensor node in the $j^{th}$ cluster.	$TN_{sum,j}$	The time spent on collecting data of neighboring clusters of the $j^{th}$ cluster
$B_{i,t}^{j}$	The residual energy of the $i^{th}$ sensor node in the $j^{th}$ cluster.	$O_{ch\arg e}$	The optimal charging order of the clusters.
$U_{i,j}$	The charging rate of $i^{th}$ sensor node in $j^{th}$ cluster.	$weight_{i,j}$	The weight value of the $i^{th}$ sensor node in the $j^{th}$ cluster.
$D_{j,j+1}$	The travel distance of the MV between the $j^{th}$ cluster and $j+1^{th}$ cluster.	$NE_{j}$	The set of neighboring clusters of the current $j^{th}$ charging cluster
$\tau_{path,j}$	The travel time of the MV between the $j^{th}$ cluster and $j+1^{th}$ cluster.	$weightU_{c,j}$	The weight value of the $j^{th}$ cluster during the UAV data collection.
ξ	Even time slot.	$R_{ava}$	The average packet arrival rate of CHNs.
$\eta_{i,j}$	The normalized dead time of the $i^{th}$ sensor node in the $j^{th}$ cluster.	$C_{3}$	The set of clusters which send data to the UAV.
$v_{c}/v_{u}$	The speed of the MV or the UAV.	$CH_{j}$	The cluster head node of the $j^{th}$ cluster.
α	The coefficient of battery capacity.	l	The coefficient representing the path condition.

**Table 2:** A list of abbreviations.

MV	Mobile vehicle
UAV	Unmanned aerial vehicle
WSN	Wireless sensor network
WRSN	Wireless rechargeable sensor network
WET	Wireless energy transfer technology
CDV	“Collect data vehicle”
EJCCA	Efficient joint charging and collecting algorithm
EDCA	Efficient data collection algorithm
CHN	Cluster head node

**Table 3:** Different values of ϖ,θ,ϑ.

	Set	1	2	3	4	5	6	7	8
Factors									
ϖ		0	0.2	0.33	1	0.5	0.4	0.4	0
θ		0	0.4	0.33	0	0.3	0.4	0.5	1
ϑ		1	0.4	0.34	0	0.2	0.2	0.1	0

## Data Availability

Codes of our proposed algorithm is available in the link: https://blog.csdn.net/qq_42031990/article/details/116008390.
